# Towards developing robust solid lubricant operable in multifarious environments

**DOI:** 10.1038/s41598-020-72666-4

**Published:** 2020-09-21

**Authors:** Aditya V. Ayyagari, Kalyan C. Mutyala, Anirudha V. Sumant

**Affiliations:** Center for Nanoscale Materials, Argonne National Laboratory, Lemont, IL 60439 USA

**Keywords:** Materials science, Nanoscience and technology

## Abstract

Conventional solid lubricants such as MoS_2_, graphite, or diamond-like carbon films demonstrate excellent tribological performance but only in specific environments due to their inherent materials properties. This limitation prohibits using these solid lubricants in environments that change dynamically. This study presents the results of a novel solid lubricant that was developed using a combination of solution-processed 2D-molybdenum disulfide and graphene-oxide (GO) that can be deposited on to stainless steel substrates using a simple spray-coating technique and show exceptional performance in multifarious environments namely, ambient (humid) atmosphere, dry nitrogen, and vacuum. The tribological performance of the coatings was evaluated using a ball-on-disc sliding test and demonstrated an excellent wear/friction performance in all environments and coating survived even after 44 km of linear sliding. Transmission electron microscopy and Raman spectroscopy analysis of the tribolayers suggested in-operando friction-induced re-orientation of MoS_2_ layers that were protected by GO layers and, an absence of MoO_x_ peaks indicate a strong resistance to intercalation with moisture and oxygen. The simplicity and robustness of the hybrid MoS_2_–GO solid lubricant in mitigating wear-friction behavior of steel-on-steel tribopair in a multifarious environment is a game-changing and is promising for various applications.

## Introduction

The specificity of environments that permit an optimum performance of solid lubricants is an engineering limitation that prohibits a wide-scale adaptation of ‘green-technologies’ in several dynamic engineering systems. It has been broadly established that graphite and carbonaceous materials perform relatively better in humid environments^1–9^ and MoS_2_ and other layered transition dichalcogenides in oxygen-free dry environments since intercalation rapidly deteriorates the lubricity properties^10–20^. Specific to MoS_2_, material modification strategies have been proposed to enhance the performance of the lubricant coatings by delaying kinetics of degradation^15,16^. Processing techniques that protect layered 2D materials by sealing the lubricant from deleterious environments has been a predominant path to delay intercalation and thereby extend service life. In MoS_2,_ the intercalation resistance could be achieved by either macroscale layered composite structure or by flake level encapsulation. For example, Voevodin et al.^21^ developed chameleon coatings that were essentially hybrid magnetron assisted pulsed laser depositions (MSPLD) of Al_2_O_3_/DLC/Au/MoS_2_. Similar examples of binary and tertiary composites such as MoS_2_/Ni; MoS_2_/Ti; MoS_2_/Sb_2_O_3_; Mo_2_N/MoS_2_/Ag; MoS_2_/Au/Sb_2_O_3_ and MoS_2_/C/Sb_2_O_3_ that have been shown to significantly lower friction and wear for up to 10,000 cycles^22–24^. These lubricating coatings were reported to be functional between various tribopairs and temperature-humidity conditions. On the other hand, layer level encapsulation/composites have also been demonstrated to be equally effective. Zhang et al.^25^ have demonstrated the lowering of friction by developing a hybrid MoS_2_–rGO structure via chemical synthesis route. Friction recorded against a metal matrix composite with the hybrid lubricant and commercial alumina ceramic ball was in the range of 0.21–0.35 over a broad range of temperatures. In a different work, Chen et al., used a surfactant-assisted hydrothermal route to fabricate MoS_2_/rGO hybrids to achieve a maximum level of MoS_2_ and graphene incommensurate interface. The lowest recorded friction was 0.09 with monolayer hybrids while friction was observed to be slightly higher with multilayer structures^26^. Su et al.^27^ have proposed a hydrothermal route where MoS_2_ was protected from the environment by in situ synthesis into the Al_2_O_3_ matrix. This approach was resulted in lowering the friction from 0.74 to 0.19. The DC Magnetron sputtering technique was used to develop MoS_2_–C layered coatings with distinct boundaries^27,28^. It was observed that the carbon phase not only improved the mechanical properties of the coating but also improved the wear/friction performance by inhibiting the oxidation of MoS_2_. A brief survey of the composites, process modifications and other mechanisms adopted to enhance the tribological performance of MoS_2_ have been summarized in Table 1.

**Table 1:** Summary of MoS_2_ derived solid lubricant materials and their tribological properties.

Sl #	Lubricant	Coating technique	Disc	Counterface	Normal load/contact pressure	Sliding speed	Cycles/distance/duration	Friction coefficient	
1	Al_2_O_3_/DLC/Au/MoS_2_^15^	Hybrid magnetron assisted pulsed laser deposition	440C steel	M50 steel balls	100 g (translating to ∼ 0.5 GPa)	0.2 m/s	10,000 cycles	0.02–0.03	In Dry N_2_
								0.10	Humid air
								0.10	At 500 °C
2	Ni–W–MoS_2_^21^	Reverse pulse plating	Carbon steel	Stainless Steel	6 N (1.187 GPa)	1.03 cm/s	2,955 cycles	0.1–0.3	
3	MoS_2_/Au/Sb_2_O_3_^24^	Pulsed laser deposition	304 stain-less steel	304 stain-less steel pin	5 N (1.07 GPa)	10 mm/s	10,000 cycles	0.028	
	MoS_2_/C/Sb_2_O_3_^24^							0.034	
	MoS_2_/Sb_2_O_3_^24^	DC Sputtered						0.043	
	MoS_2_/Ti^24^	DC magnetron sputtering						0.055	
	MoS_2_/Ni^24^							0.084	
4	Mo_2_N/MoS_2_/Ag^23^	Magnetron sputtering	440C steel	Alumina	1 N (0.739 GPa)	0.2 m/s	10,000 cycles	0.56	
5	MoS_2_/rGO^26^	Hydrothermal route	Steel	GCr15	5 N (1.26 GPa)	100 rpm		0.09	
6	Alumina-MoS_2_^27^	Sintering	Alumina composite	Si_3_N_4_	5 N	5 cm/s	2 h	0.19	
7	MoS_2_/rGO^25^	Modified Hummer method	Fe–Ni matrix self-lubricant composites with	Alumina	10 N	0.08 m/s	15 min	0.21–0.35	
8	MoS_2_–C^29^	Magnetron sputtering	High speed steel	GCr15	10 N (0.91 GPa)	5 Hz	2 h	0.050 -0.100	
9	WS_2_–MoS_2_^30^	Radio frequency sputtering	440C steel	440C steel	3 N (0.25 GPa)	0.52 m/s	8 × 10^5^ cycles	0.05	
10	MoS_2_–GO^[This work]^	Sonix-coating	440C	440C	1, 3, 5, 7 9 N (0.48–0.99 GPa)	0.1 and 0.5 m/s	0.5–44 km	0.11–0.08 in ambient air and 0.14–0.03 in Dry N_2_ (present work)	

**Figure 1:**
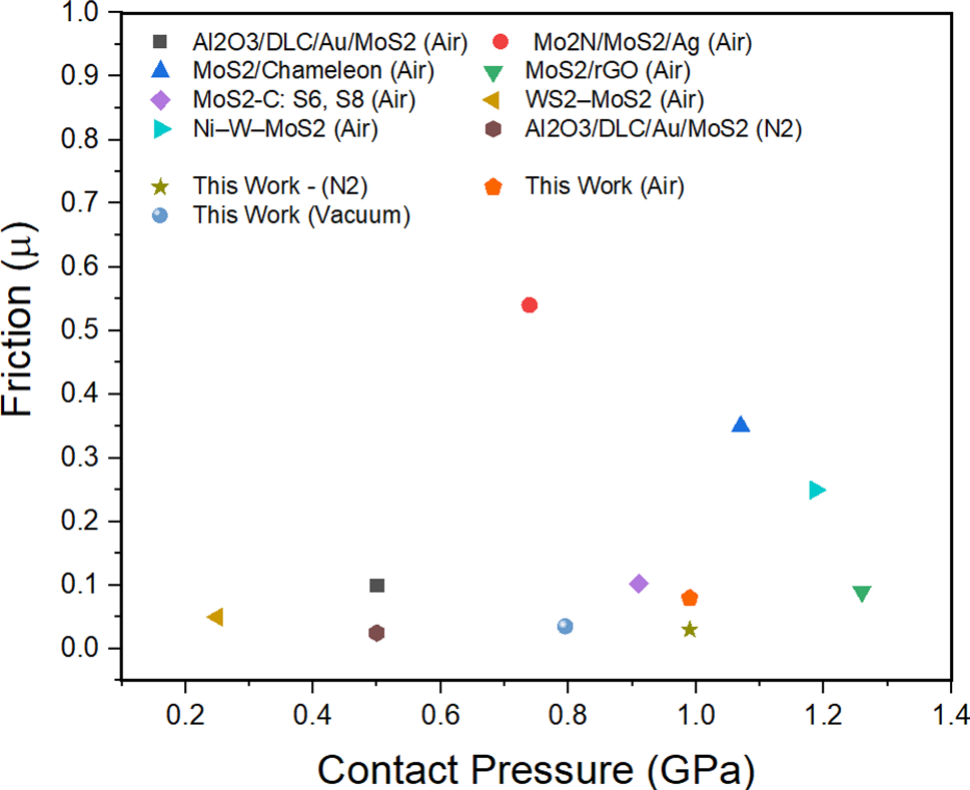
Summary of various of MoS_2_ derived composites/coatings developed and the resulting friction observed as a function of the normal load.

It can be noted that some of these lubricants also have performance limitations under high contact pressures and prolonged sliding distances. A graphical summary of the lubricants shown in Table 1 is shown in Fig. 1. demonstrating superior performance of the present MoS_2_–GO materials based Sonix coating in dry nitrogen, air and in vacuum environments as compared to other MoS_2_-based coatings at high load and high sliding velocity and have shown much lower wear rates than other MoS_2_-based coatings.

The realization of the full potential of solid lubricants is also stunted due to significant commercial factors, such as laborious and cost intensive processing steps that involves vacuum deposition techniques such as sputter deposition; pulsed laser deposition; and carefully controlled hydrothermal routes. Also, the aforementioned techniques were predominantly developed for specific applications such as in the aerospace industry and may not be suitable for range of other typical tribological applications such as rolling element bearings, gears, camshafts, valve trains, and journal bearings. The literature survey also indicates that the materials’ design space defined by stringent boundary conditions of commercial viability, mechanical robustness, and environmental compatibility is vastly empty leaving enormous scope for further development. This work presents the results of a solid lubricant derived from 2D materials that fit well within the aforementioned design constrains. In addition to these parameters, an attempt was made to overcome three main limitations such as the requirement to have an inert counterface [such as diamond like carbon (DLC), Alumina (Al_2_O3) or Silicon–nitride (Si_3_N_4_)]; the restrictions on size and shape of the substrate to be coated due to complex CVD and PVD techniques and limitations on test loads and speeds. The lubricants were synthesized by the sonix technique and were deposited using a simple spray-coating technique in air followed by testing at high loads and high speeds with steel against steel, a most commonly used tribo-pair in bearings and many tribological applications. The wear and friction characteristics were recorded, and the observed results were explained using Raman spectroscopy and transmission electron microscopy. The robustness of the 2D lubricant along with the simplicity of the deposition technique makes this technology lucrative for several industrial applications.

## Results and discussion

**Figure 2:**
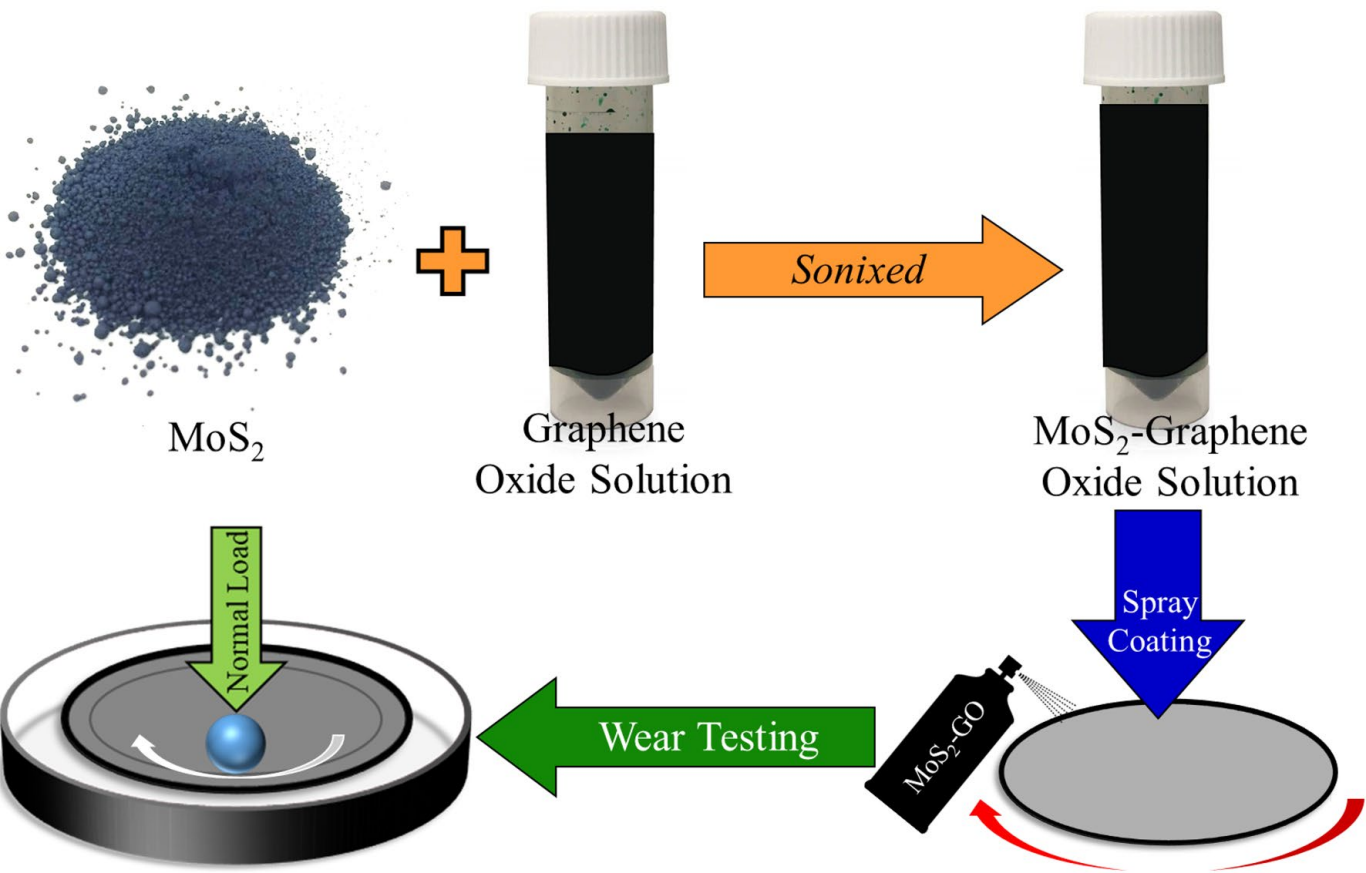
Schematic showing a lubricant spray coating process followed by pin-on-disc test.

The schematic of the novel sonixing technique is shown in Fig. 2. Sonix consists of suspending and intimately mixing MoS_2_ and Graphene oxide using ultrasonication method. The solution thus made was spray coated using a air-spray gun.

### Coating and wear/friction analysis

**Figure 3:**
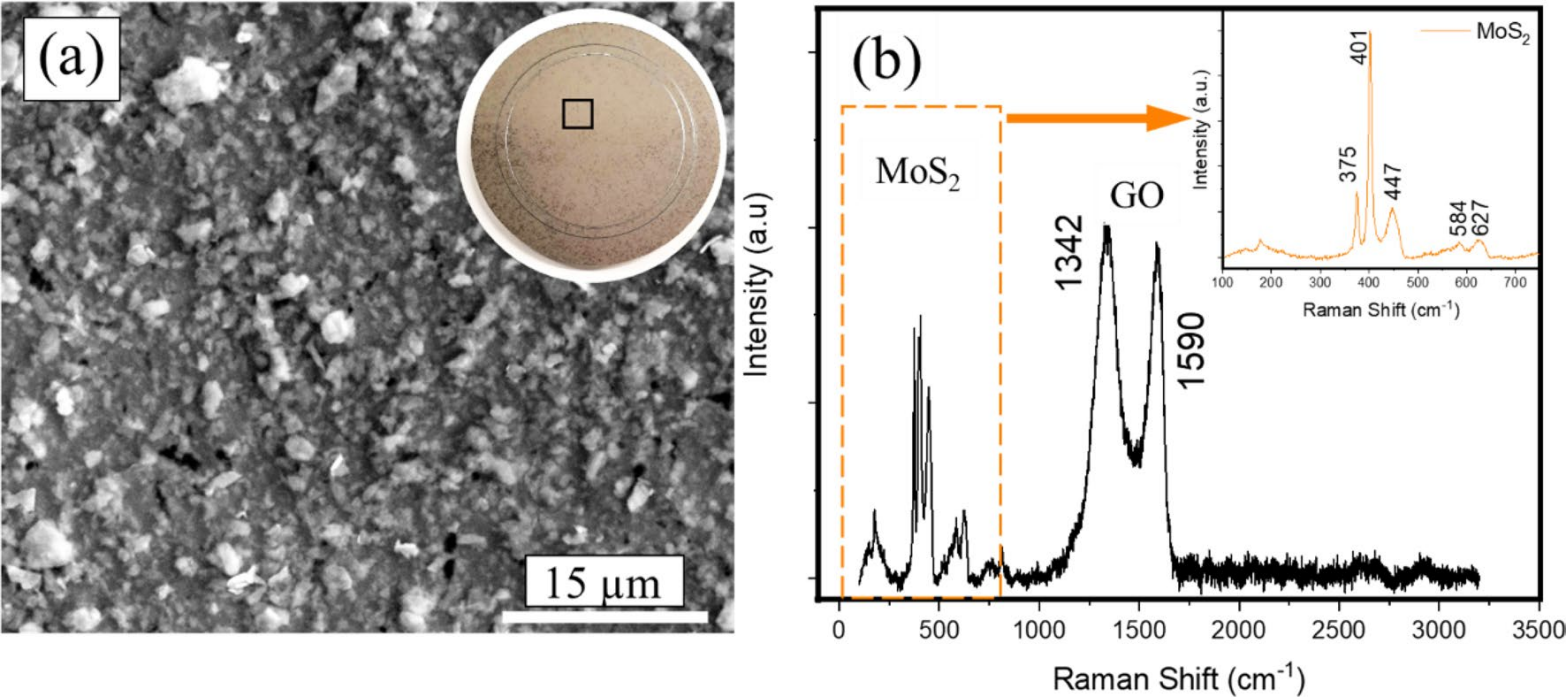
(**a**) Scanning electron microscope image of the MoS_2_–GO solid lubricant spray coated on to a steel substrate. The inset is the optical image of the lubricant coated steel disc and the area of SEM imaging (**a**) is highlighted with a box. (**b**) Raman spectra of the solid lubricant composite coating showing unaltered structures of the MoS_2_ and GO.

The surface morphology of the as-deposited coating on the steel sample is shown in Fig. 3a.

The surface shows a random mixture of the solid lubricants deposited on to the steel substrate. The Raman spectra acquired from the as-deposited samples are shown in Fig. 3b and have the characteristic signature of both MoS_2_ and GO. The spectra show the characteristic MoS_2_ peaks at 380, 401, 460, 578 and 644 cm^−1^, and graphene oxide peaks at 1342 and 1590 cm^−1^ respectively indicating that the individual materials of the lubricant retained their pristine structure (Raman spectra of the pristine materials is shown in Supplimental Material, Figure S1) and no observable chemical changes occurred due to the sonication process in the solution processing route and sonication process mostly helped in producing a uniform mixture of MoS_2_ and GO (A transmission electron microstructure of MoS_2_ embedded in graphene oxide sheets is shown in Supplimental Material, Figure S1).

**Figure 4:**
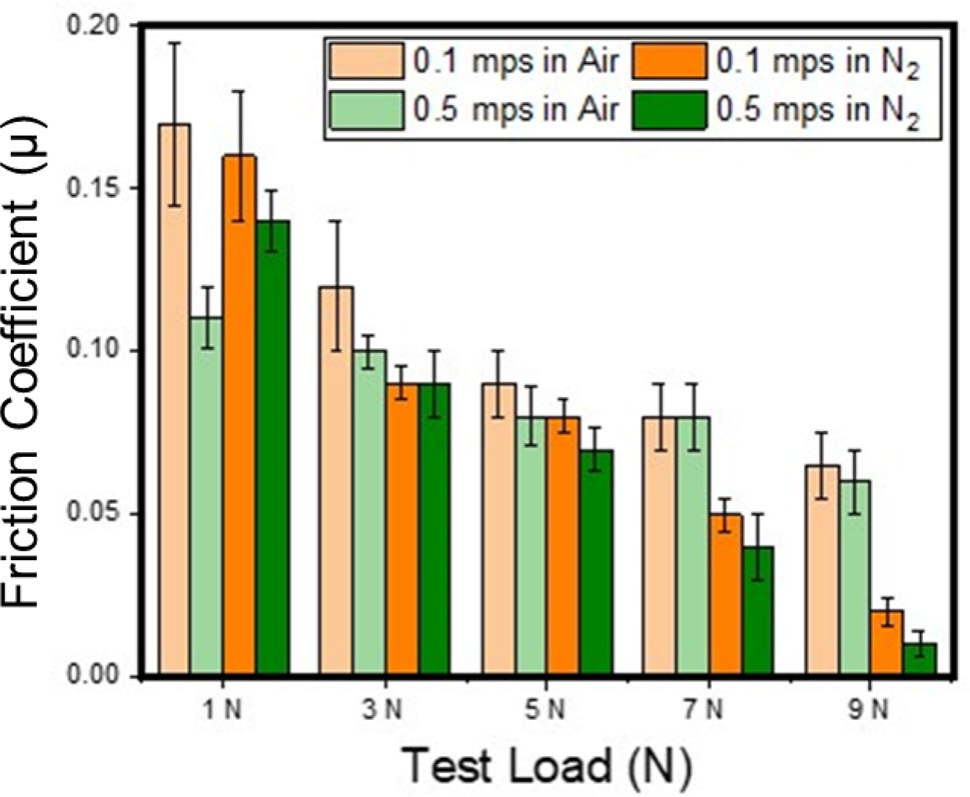
Friction coefficient as a function of load in dry nitrogen and in air environments when sliding bare 440C steel ball against 440C steel disc coated with MoS_2_–GO solid lubricant.

Before the assessment of solid lubricant performance, base-line metrics of wear and friction values were established with 440C over 440C tribopair. The steel on steel friction at 1 N load and sliding at 0.1 m/s was 0.85 ± 0.1 and is in agreement with the previous reports^31,32^. Following baseline experiments, the ability of the solid lubricant to reduce friction and wear was measured over a broad range of contact pressures and sliding speeds, and the summary of the frictograms is presented in Fig. 4.

**Figure 5:**
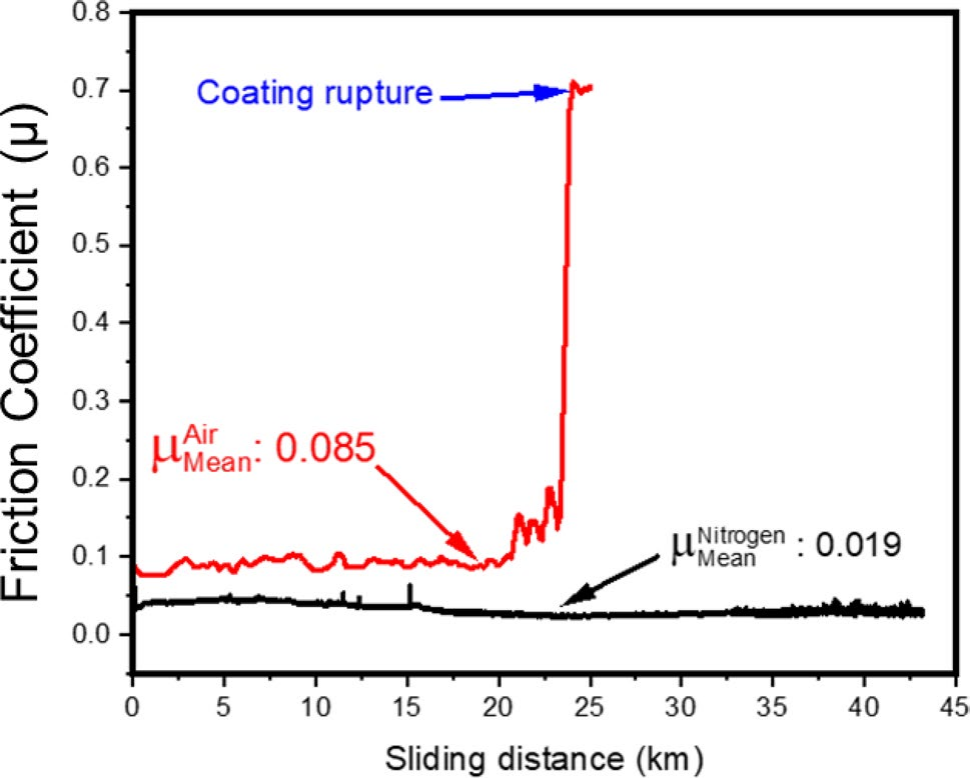
Long term endurance testing of the solid lubricant in ambient air showing removal of the coating towards the end of the test (23 km) and in dry nitrogen with no sign of degradation of the coating even after 44 km of testing.

The friction was observed to decrease with increasing load under both test environments. Friction under ambient condition ranged between 0.17 and 0.09 with the load increasing from 1 to 9 N at 0.1 m/s whereas it was 0.11 to 0.08 at 0.5 m/s. Under dry N_2_ conditions, friction observed to be between 0.16 and 0.04 when tested at 0.1 m/s speed and 0.14 to 0.03 when tested at 0.5 m/s. The data in the frictograms can be summarized as follows: lowering of friction with increasing normal load and test speed; greater longevity when tested in dry nitrogen (reduced oxygen and moisture content) with total sliding distance extending up to 44 km without any sign of failure of the coating versus up to 23 km (with failure of the coating towards the end of the test) in the case of ambient testing as shown in Fig. 5 (See Supplementary Figure S2 for full data). The standard deviation in the friction values at lower loads is relatively high as compared to the ones at 5 N and 7 N. This is due to the higher initial roughness of the as-coated / deposited surface seen in Fig. 2. The surface gets smoothened out resulting in decreases in friction with increasing normal load. The friction appears to have a large standard deviation at low friction numbers close to the superlubricity limit (0.01) which is due to the sensitivity of the load sensor.

**Figure 6:**
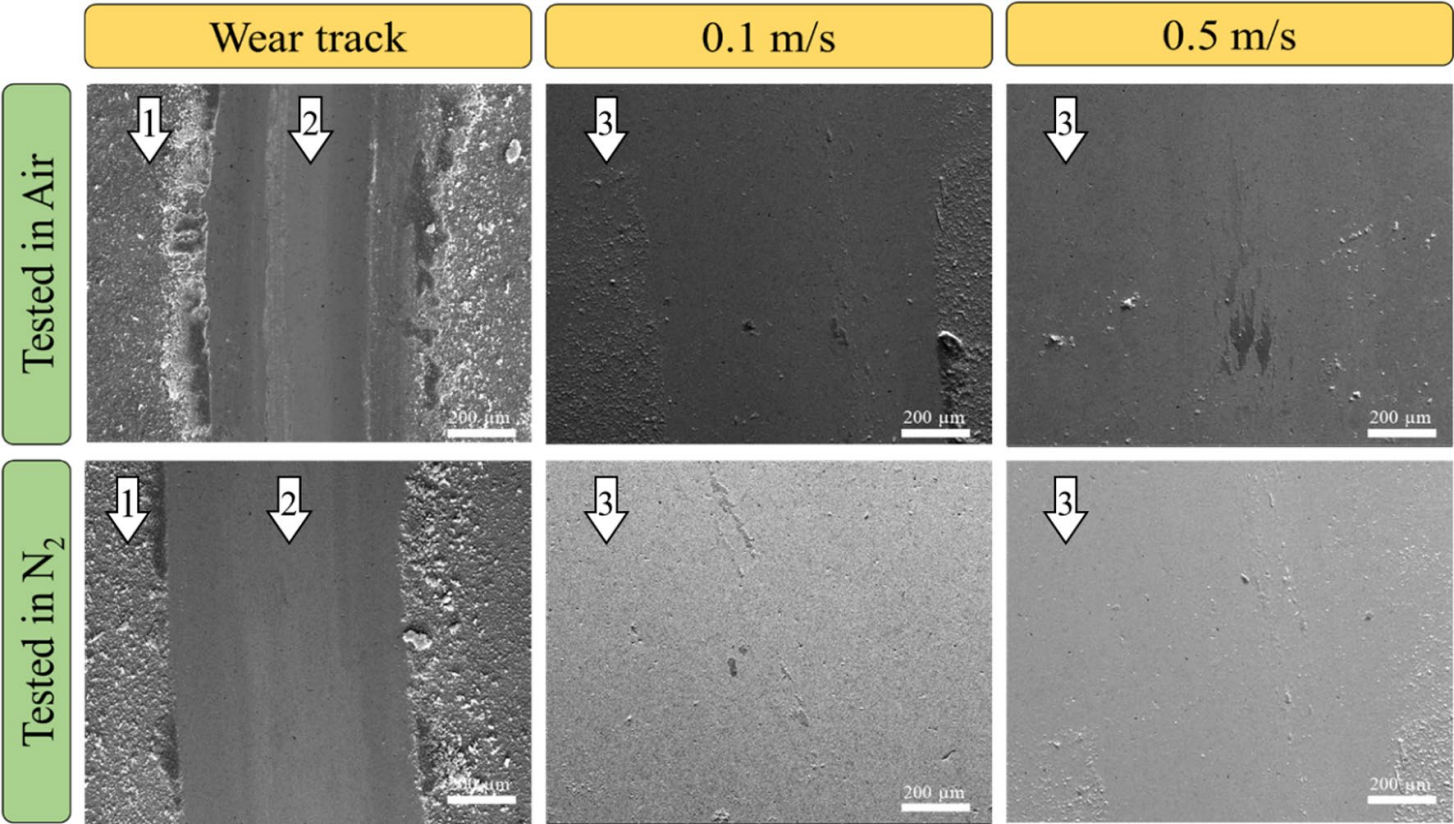
Scanning electron microscope images of wear tracks on the steel disc in both ambient and dry N_2_ conditions. The as-deposited coating on the samples tested in air and dry N_2_ are pointed with 1; the wear tracks were on the coated samples (showed with pointer 2) appeared to be wider at relatively higher loads, whereas there was no calculable wear on the underlying steel substrate as indicated with pointer arrows 3.

**Figure 7:**
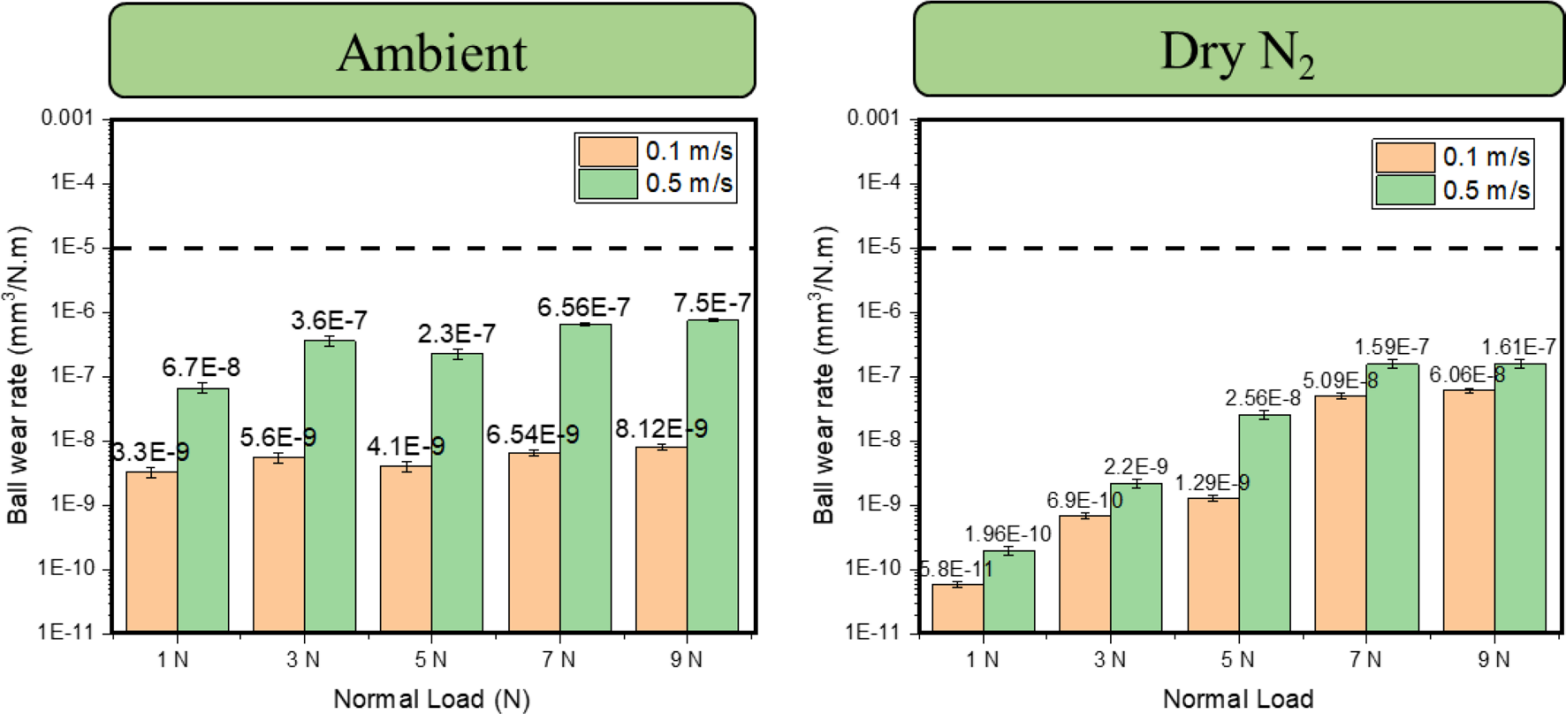
Wear rate on the counterfaces tested in ambient condition and in dry nitrogen. The dashed line is a reference steel-on-steel wear rate tested at 1 N in air and dry N_2_ respectively.

The evolution of the surface morphology and preliminary elemental analysis of the tested wear tracks on the steel discs was carried out using scanning electron microscopy coupled with EDS. EDS data (not shown here) from the respective samples tested both in ambient and dry N_2_ did not show a signature of constituent elements from the bulk steel sample indicating that the lubricant coating was relatively thick and remained intact, and effectively prevented steel-on-steel contact. This prima facie indicated that no actual wear on the steel disc occurred. To confirm this and calculate the actual wear loss, the coating was removed by washing the discs under running tap water. Observations on the underlying steel surface after stripping the coating were in line with the EDS observations, i.e., there was no wear scar or damage on the discs even after prolonged testing at high contact pressures and sliding speeds as shown in Fig. 6. The dimensions of the wear tracks on the coated substrates increased with increasing normal load, although no such effect was observed on the underlying steel disc itself. To confirm the SEM observation of no damage on the steel substrate, profiler scans were taken and interferograms were constructed which are presented in Supplemental Information Figure S3. In stark contrast, other state of the art GO based coatings were observed to show significant wear in the range of 1–30 × 10^−6^ mm^3^/N m^25,33,34^ on the discs. The estimated Archard wear rate for the respective counterface balls in both ambient and dry N_2_ conditions shown in Fig. 7. The wear rate in ambient condition although increased with increasing normal load, the rate of increase was lower compared to the dry N_2_ sliding condition. It is also, however, interesting to note that the absolute value of the wear rate was lower in the N_2_ sliding condition.

### Tribolayer formation mechanism

**Figure 8:**
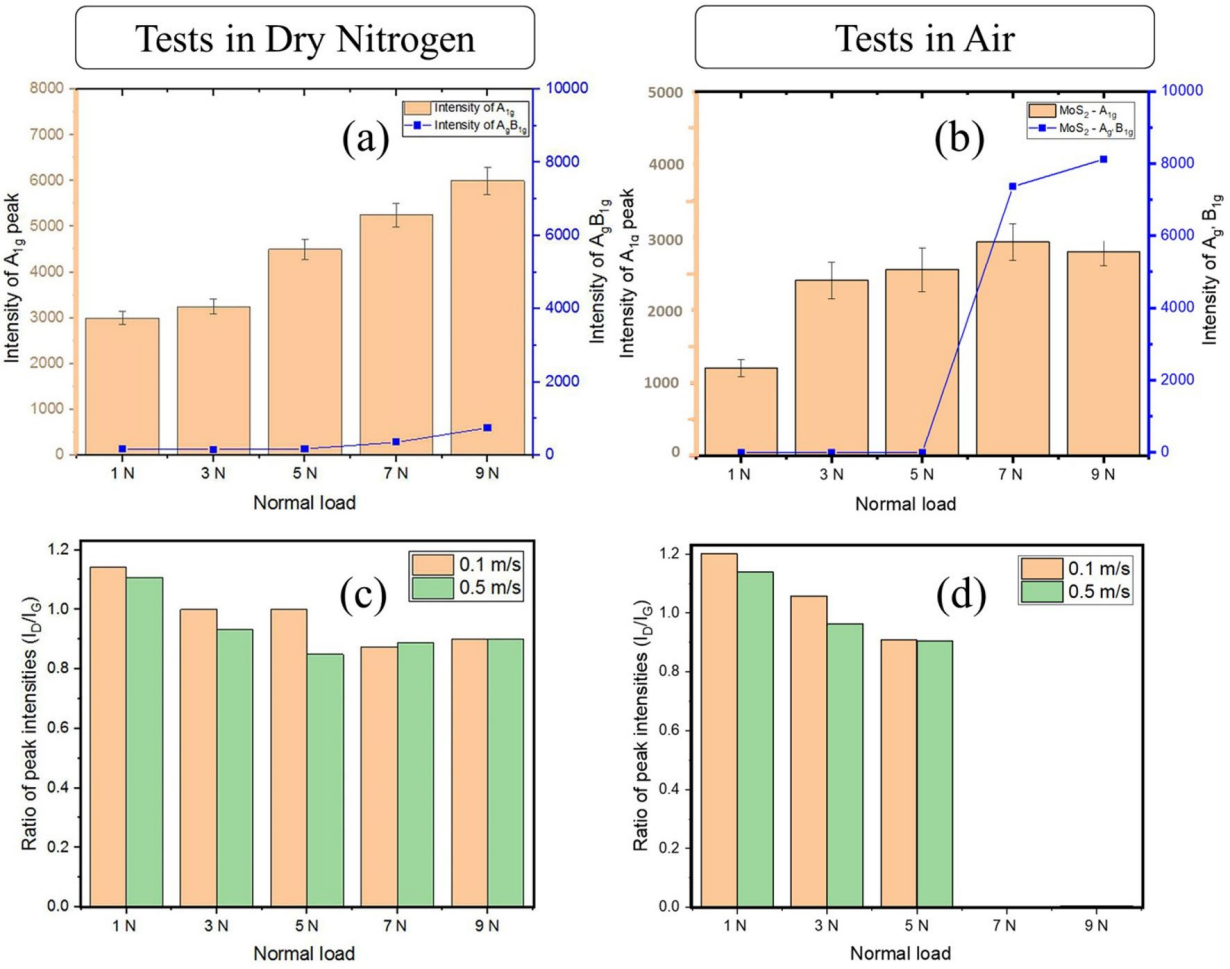
The bar chart of the Raman spectra in terms of the relative intensity of MoS_2_ and graphene oxide peaks recorded on the tribolayer of the discs. The figure (**a**) and (**c**) represents variation in the intensity of MoS_2_, MoO_x_ and D/G ratios of the GO peak for tests in the dry N_2_ and figure (**b**) and (**d**) represents the same peak features for tests in air respectively. The structural transformation of the coating material is noticed in the form of a clear trend in terms of the relative intensity of MoS_2_ and graphene oxide peaks.

The SEM/Raman observation indicates that the tribolayer formation was the key feature in the resulting excellent wear/friction properties. To further understand the mechanism of tribofilm formation, Raman spectra were acquired across the wear tracks on all samples tested in air and in dry nitrogen environments and are shown in separate figures (Supplementary Figure 4 and 5). The Raman spectra acquired from the samples tested in dry nitrogen did not show any effect of normal load and sliding speed and remains unchanged (Supplementary Figure 4). In stark contrast, multiples changes were observed in the samples tested in air (Supplementary Figure 5). The Raman spectra from the coatings on discs are characterized by few important features of MoS_2_ and GO peaks respectively. These delineate the lubrication mechanism and structural transitions taking place in the tribofilm and they are collectively displayed in the following Fig. 8a–d in terms of bar charts. Firstly, the intensity of MoS_2_ peaks (mention MoS_2_–A_1g_) increased with increasing contact pressure under both sliding velocities as shown in Fig. 8a (dry nitrogen) and Fig. 8b (air) indicating grain growth and reorientation. Secondly, the 666 cm^−1^ (mentioned MoS_2_–A_g_–B_1g_) and other MoO_x_ characteristic peaks intensities were relatively stable until 5 N and a sligt increase in the peak intensity was observed for 7 N and 9 N load in dry nitrogen tests (Fig. 8a) but in case of air, a significant increase in these peak in tensities were observed for 7 N and 9 N load(Fig. 8b). The peak at 666 cm^−1^ corresponds to the Mo trioxide compounds (MoO_3_), which indicates that MoS_2_ was structurally stable up to loads reaching 5 N, but slowly started to form oxides at 7 N and beyond when tested in air. This is an important differentiating aspect in the air-tests as compared to the test conducted in Dry N_2_. It goes to indicate that MoS_2_ does intercalate and convert into its oxides in air when tested at very high loads and high speeds, albiet its robustness is uncompromised at loads upto 5 N. One can also look at the D/G peak intensity ratio for the GO in case of dry N_2_ and in air as shown in Fig. 8c,d respectively to understand the degradation of GO. The ratios of intensity of D and G peaks remained nearly similar when tested in Nitrogen, whereas, it decreased with increasing load and sliding velocity and finally, a complete absence of GO peaks at 7 N and 9 N when tested in air. This indicates a gradual degradation of GO as contact pressure and sliding velocity was increased and eventually degradation of GO from the wear track completely in case of air test as seen from Fig. 8d (and corresponding Raman spectra shown in the supplementary Figs. 5).

These results collectively indicate that in operando tribochemical changes played a significant role in the structural evolution of the tribolayer, and thus have resulted in lowering the friction. These changes in conjunction with frictograms (Fig. 4 and Supplementary Figure 2), clearly show that lubricity improved with increasing load and speed for both test conditions(dry N_2_ and in ambient air) but with extended life-time when tested in dry N_2_. Raman spectra for all test conditions can be found in Supplemental Fig. S4 and Fig. S5 for more details.

It is hypothesized that increased energy imparted at the sliding interface due to increasing normal load and/or sliding velocity accelerates the process of smoothening the coating surface, due to which friction further lowered as seen in the frictograms. Increasing intensity of MoS_2_ peak suggests that the smoothening effect is accompanied by friction-induced crystallographic re-orientation of MoS_2_ flakes in the sliding direction that resulted in a pronounced Raman peak. The re-orientation of the MoS_2_ flakes can also be identified from the increasing intensity of the A_1g_ peaks as a function of the normal load in Fig. 8a. Upon increasing the energy input to further higher levels (as in 9 N and 0.5 m/s velocity), there might have been sufficient energy input in accelerating surface smoothening and re-orientation phenomena. Additionally, encapsulation of MoS_2_ by GO flakes may have helped to reduce the poisoning effect of oxygen and moisture on sulfur and thus enhancing recrystallization and re-orientation of MoS_2_ basal planes. A wider lubricious contact surface composed of highly oriented MoS_2_ flakes may explain the lowering of friction with increasing test loads. Diminishing intensity of Graphene Oxide peaks may suggest that it is actively transforming by rupture and consequently forming new dangling bonds. This in conjunction with decreasing I_D_/I_G_ ratios indicating that the two-phase transformations occurring in the GO are: first, the deterioration of GO which is represented by the overall lowering of D and G peak intensity, the second is the formation of more defective graphitic carbon phase due to repeated rubbing and re-deposition as reflected by the increase in the D peak intensity. Since the two phenomena are taking place simultaneously and become pronounced with increasing load, it may be deduced that the reaction-path precedes by the deterioration of GO, followed by more defective graphitic carbon transformation. A consequence of deterioration/breaking of GO sheets is the generation of a large number of dangling bonds that have a greater affinity towards H and OH ions in the vicinity. Formation of graphitic carbon phase that characterized by the presence of a large number of dangling bonds, that potentially attracts H and OH must have contributed to friction reduction^4,5^. It is well known that the tribological performance of graphene/graphite is better in humid conditions than in inert/dry N_2_ atmospheres due to chemisorption of the of vapor molecules at the nascent edges^4–6,14,35–41^. Preferred chemisorption of hydroxyl groups by the nascent dangling bonds in graphitic carbon may have effectively prevented interaction between Mo and Oxygen. This is seen from the Raman spectra extracted from the initial test conditions (Fig. 8a) wherein peaks corresponding to MoO_x_ (285, 666, 820 and 995 cm^−1^^39^) are absent, indicating high intercalation resistance.

The threshold of maintaining the intactness of the coating is observed to overcome at the 7 N load condition, where the signature of defective graphitic carbon was begins to disappear alongside the appearance of the MoO_x_ peak at the 666 cm^−1^ peak position. This is seen as the steep rise in the A_g′_B_1g_ intensity in Fig. 8a and the complete absence of datum points 7 and 9 N in Fig. 8b. This effect has seen to be more pronounced for tribotesting in ambient conditions (Supplementary Figure 5) as MoO_x_ peaks are significantly higher in intensity as the load increases to 7 and 9 N and the G peaks in GO completely disappears with a sharp increase in the D peak intensity. The deterioration of GO has a direct consequence on the life-time of the coating as can been seen from endurance testing shown in Fig. 5. However, the effect of deterioration of the coating does not immediately appear to have a response on the friction values as seen from the frictograms (Supplementary Figure S2). This may arise from the fact that although the signatures of the Raman peaks are changing and chemical compounds there are evolving, the film coating remained intact on the surface. The effect of the changes in the tribolayer does not become obvious until much longer sliding duration/distance as seen from the long term test in Fig. 5. The low friction values are maintained well beyond the 500 m marks in the prolonged sliding test after which actual rupture and friction spikes are observed.

**Figure 9:**
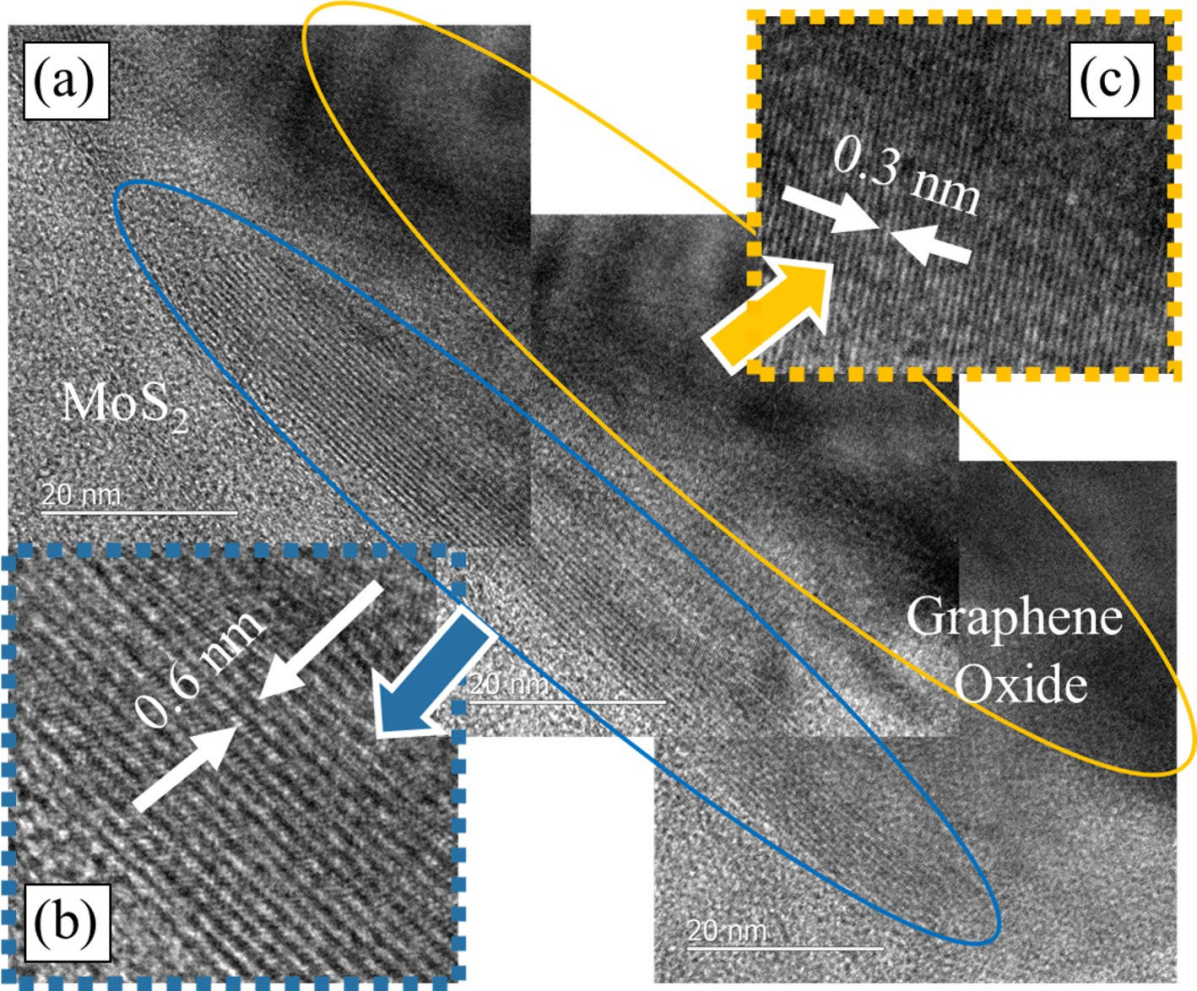
Transmission electron microscope image of the lubricant showing graphene-oxide and MoS_2_ in the layer formation. (**a**) Montage of MoS_2_ layers underneath graphene-oxide.

From a structural point of view, it has been previously shown that highly oriented MoS_2_ flakes are inherently more resistant to oxidation^40^. In the present case, the encapsulation of MoS_2_ flakes by graphene oxide may have helped to grow the basal planes of MoS_2_ to a larger length (as seen from the TEM image shown in Fig. 9a) since the formation MoO_3_ was restricted as evident from the Raman characterization.

**Figure 10:**
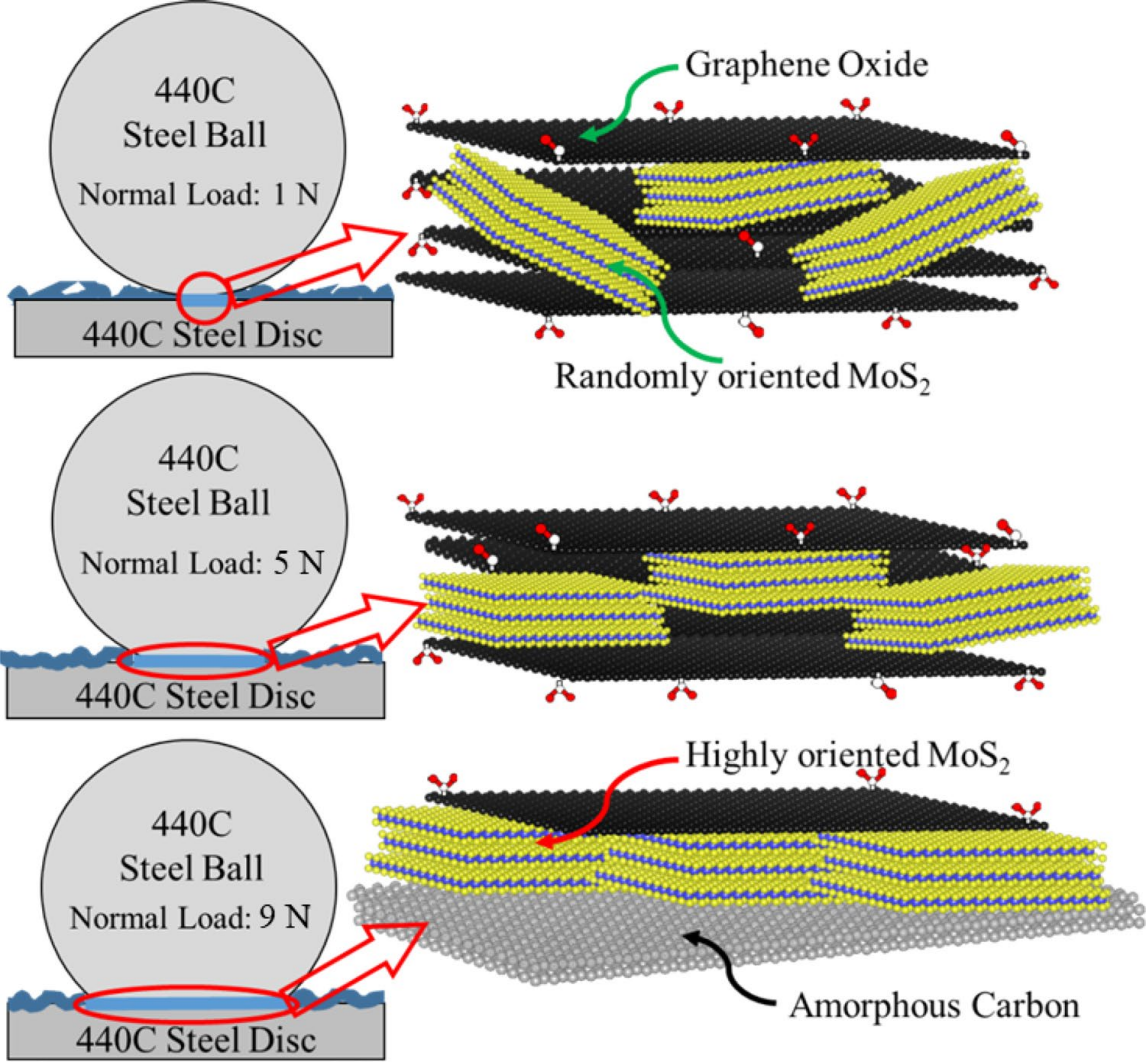
Schematics showing the evolution of tribolayer and with increased energy input into the system.

Figure 10 shows the mechanistic evolution of the tribolayer with increased energy input to the system that facilitates encapsulation and friction-induced re-orientation of MoS_2_ basal planes in the sliding direction, which finally resulted in lowering friction and wear significantly. This observation is in line with previous reports (mostly in dry atmosphere or in vacuum) where lowering of friction with time (known as run-in) was correlated with friction-induced crystallographic re-orientation of the MoS_2_^35,41^, whereas in the current investigation, the combined effect of load and sliding speed were also observed to accelerate the transitioning into steady-state low friction regime even in multifarious environments.

To confirm the aforementioned hypothesis TEM samples were extracted from the tribolayers of the three load conditions. The samples from lower test loads and speeds did not show any significant layer level bonding and phase re-formation and largely maintained their typical characteristics. This drastically changes to form a robust tribolayer as shown in Fig. 9 (at high loads and high sliding speeds under both environments), wherein multiple layers of MoS_2_ have been observed to be stacked and oriented to form a long packet, while intact graphene oxide was observed on one side and amorphous carbon on the other side of the packet, indicating that graphene oxide indeed disintegrates to some extent as well as forming layer level encapsulation. The TEM observations corroborate well with the operando tribochemical evolution as seen in the Raman spectra.

**Figure 11:**
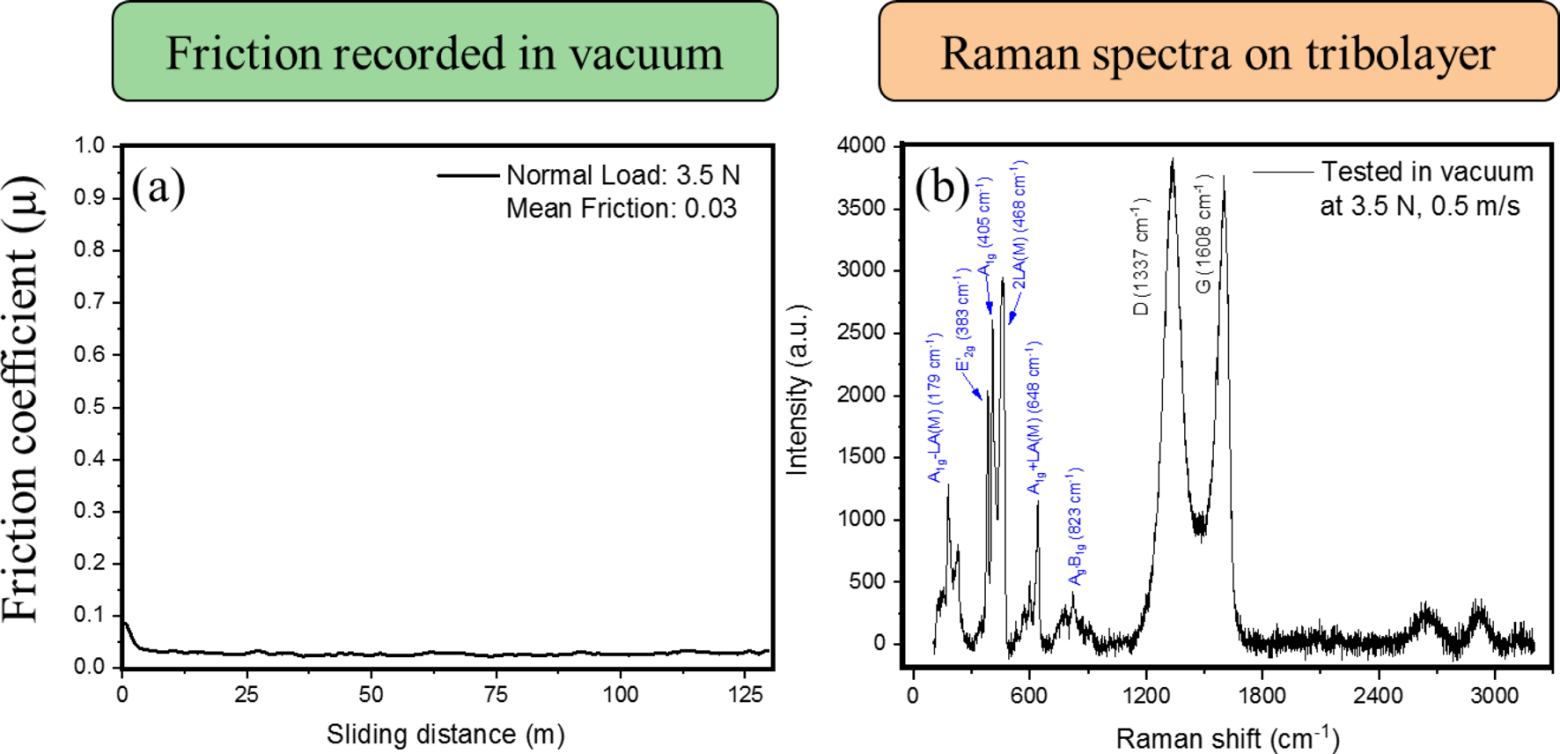
Friction recorded in a vacuum (**a**) and Raman spectrum taken from the wear track on disc indicating no change from the as-deposited coating.

To truly ascertain the ability of the lubricant to perform well in multifarious environments, the coating was tested in vacuum test conditions as well. Wear experiments were carried out in high vacuum (3 × 10^−5^ Torr) at 3.5 N (upper limit of vacuum chamber load cell) and 0.5 m/s sliding velocity. The friction observed under the vacuum condition was similar to the dry nitrogen condition averaging at 0.03 (Fig. 11a). The ball wear rate was 3.5 × 10^−7^ mm^3^/N m while the Raman signature (Fig. 11b) remained relatively unchanged compared to the base material. This indicates that the coating can perform equally well in ambient, dry nitrogen, and vacuum conditions.

The current observations also push the understanding of MoS_2_’s behavior in terms of processing–structure–property relationships towards robust industrial applications. The synthesis and deposition of MoS_2_–GO using a liquid phase just as a carrier media is the prime novelity of this study, in comparision to previous reports where either MoS_2_, GO or a combination were used in oil based suspensions^42–46^. Although the orientation of individual MoS_2_–GO flakes was completely stochastic due to the nature of the spray coating technique, the encapsulation at layer-level must have been very effective that prevented coating degradation when tested in ambient atmospheric conditions at high loads and sliding speeds. Despite the current data being from ambient condition experiments, the wear-friction data (Figs. 4, 7) and run-in durations were comparable/or better than similar coatings tested in inert gaseous or vacuum conditions. The conventional CVD and PVD-based thin film coating techniques mentioned in Table 1 provide a significantly higher degree of process control in terms of structure and orientation of the MoS_2_ basal planes grown on to the substrate, however, maintaining those conditions at higher coating thickness is difficult. In contrast, the spray-coating technique, although results in random orientation of flakes, was observed to result in friction values that improved with sliding and possess excellent longevity as seen from the extended duration test shown in Fig. 5. A major advantage of the approach consisting of the simplified coating process and effective materials’ is the ease of adoption by the industry and the ability to perform without compromise across multifarious environments. Spray coating is favorable since it is easily scalable to a large area, enables ease of thickness control, is a room-temperature ambient atmosphere process, has low cost and the deposited coatings have been observed to have chemical stability. This is besides other advantages of virtually no wear on the substrate, ease of removing the coating after application may find ready application in cold metal forming industries.

## Conclusions

Solution-processed MoS_2_–GO coatings spray coated on to 440C steel substrates were evaluated for their tribological performance under ambient atmospheric conditions, dry nitrogen, and in high vacuum at high contact pressures and sliding velocities. The coatings consistently exhibited low friction, excellent wear resistance, and low run-in intervals over a broad range of test conditions. Friction was observed to evolve with time by virtue of smoothening of coating roughness followed by friction-induced crystallographic reorientation of MoS_2_ flakes with increasing energy imparted to the tribosystem. The coating was observed to be virtually inert to the effects of intercalation from oxygen and moisture at high contact pressures and independent of the atmosphere in which it is used. TEM results suggest that this was due to the protective GO encapsulation over the MoS_2_ flakes. The combination of the 2D hybrid lubricant materials and simple spray coating process makes this technology readily adaptable by the industry and presents a game-changing scenario in the utilization of 2D hybrid materials based solid lubricant that could be useful for a variety of application in the lubrication industry.

## Experimental

The solid lubricants were synthesized by a one-step mixing of 40 mg of ultrafine nano-crystalline MoS_2_ powder with 8 mL of water-based highly concentrated graphene oxide paste under ultra-sonication agitation referred to as Sonix technique. Both the materials were obtained from Graphene Supermarket. The resulting solution was diluted to 40 mL to produce a solution amenable for spray-coating. The effective concentration of 2D materials was 2 g/L of MoS_2_–GO suspended in ethanol. The solution containing MoS_2_–GO was spray coated on to a 50 mm diameter 440C steel having a roughness of Ra = 0.11 ± 0.01 µm. Approximately 3.5 mL of solution was consumed to coat the 50 mm diameter surface with a uniform coating thickness of 2.5 ± 0.22 µm, with the roughness of the as-coated being 0.88 ± 0.12 µm. The nitrogen pressure for the spray coating process was maintained at 0.5 atm to produce a fine spray-mist such that the ethanol in the solution evaporated immediately upon contact with the steel disc depositing the 2D solid materials, effectively serving the role only as a carrier. A schematic of the lubricant synthesis and coatings process is shown in Fig. 2.

The tribological performance of the dry steel discs was evaluated using Rtec multifunctional tribometer with an in-line white light interferometer. A 10 mm counterface ball of 440C stainless steel was used for ball-on-disc tests in unidirectional sliding mode. The tests were carried out at 1 N, 3 N and 5 N, 7 and 9 N correspondings to contact pressures of 480.3 MPa, 692.8 MPa, 821.4 MPa, 918.9 MPa, and 999.1 MPa respectively. The endurance of the coating was evaluated at the highest test load and sliding velocities: 9 N and 0.5 m/s. The experiments were carried out in an ambient atmosphere (RH ~ 22%), and in dry-nitrogen (dew point of − 43 °C). Sliding time was adjusted to maintain a total sliding distance of 500 m for all tests. Each test was repeated at least thrice for statistical confidence. Friction was recorded during the tests with a high-sensitivity capacitive load sensor and wear volume loss was assessed using the inline white light interferometer. The wear on the counterface was assessed by measuring the diameter of the ball cap (d) and substituting the radius of the ball (r) into the equation

1 $$ {\text{V}}_{{\text{w}}} = \left( {\frac{{\uppi {\text{h}}}}{6}} \right)\left( {\frac{{3{\text{d}}^{2} }}{4} + {\text{h}}^{2} } \right) $$

where h is the height of the ball cap calculated as

2 $$ {\text{h}} = {\text{r}} - \sqrt {{\text{r}}^{2} - \frac{{{\text{d}}^{2} }}{4}} $$

Following calculation of absolute wear loss on the counterface balls, the dimensional wear coefficient (k) was assessed by substituting the volume loss into Archard’s wear equation to as

3 $$ {\text{k}} = { }\frac{{{\text{V}}_{{\text{w}}} }}{{{\text{ N}} \cdot {\text{S}}}}{ }\left( {\frac{{{\text{mm}}^{3} }}{{{\text{N}}\,{\text{m}}}}} \right) $$

where V_w_ is the wear volume loss in mm^3^ calculated from Eqs. (1) and (2), N is the normal load in Newton and S is the total sliding distance in meter^47^. FEI Nova 600 NanoLab electron microscope was used for imaging the wear tracks. JEOL 2100F field emission transmission electron microscope was used for the morphological observations of the composite structures. Raman spectroscopy of the coatings and the tribolayer was carried out (Renishaw Raman Microscope using the red laser source (λ_LASER_ = 633 nm) and 10 × objective) to analyze the chemical state of the coating and the tribolayer formed after wear testing.

## Supplementary information

Supplementary Information.
