# Supplementary material for: Towards developing robust solid lubricant operable in multifarious environments
Source: Sci Rep. 2020 Sep 21;10:15390. doi: 10.1038/s41598-020-72666-4 (PMC7506011; doi:10.1038/s41598-020-72666-4)
Supplement: Supplementary file 1 — Supplementary Information. [file 41598_2020_72666_MOESM1_ESM.docx]

**Towards developing robust solid lubricant operable in multifarious environments**

Aditya V. Ayyagari, Kalyan C. Mutyala, and Anirudha V. Sumant*

Center for Nanoscale Materials, Argonne National Laboratory, Lemont, IL 60439

*Corresponding author: sumant@anl.gov

**Supplementary Information**

**
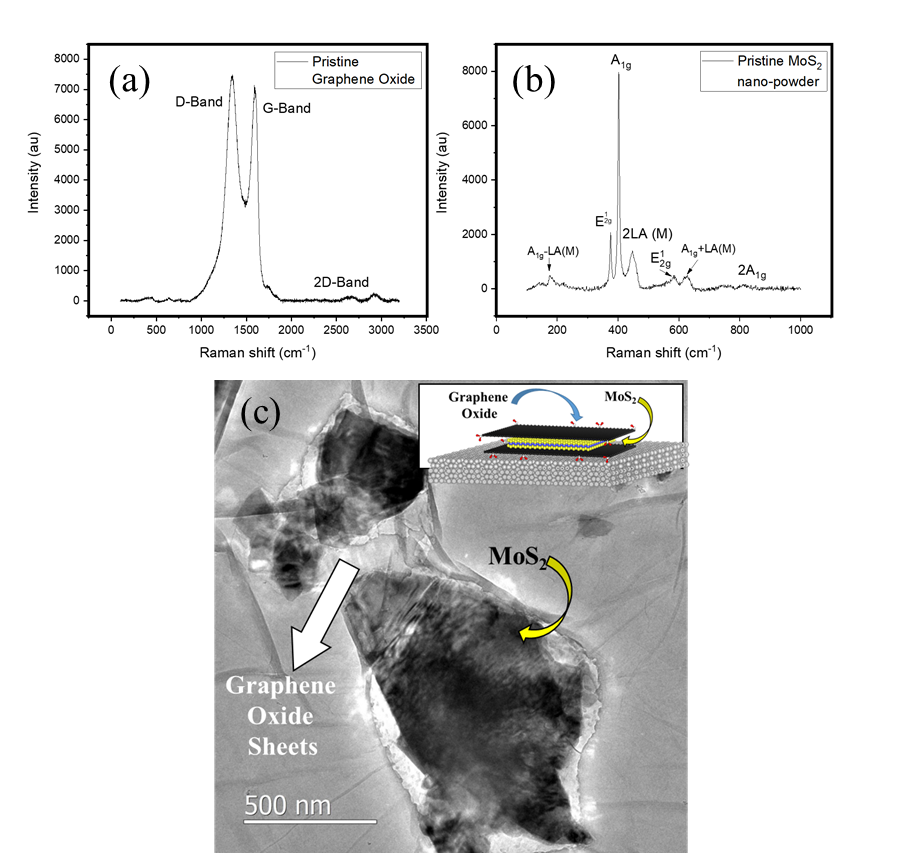
**

Supplemental Figure 1: Raman Spectra of pristine (a) Graphene Oxide (b) nano-flakes MoS_2_ (c) Transmission Electron Microscope image of as-deposited phase mixture of Graphene Oxide and MoS_2_.


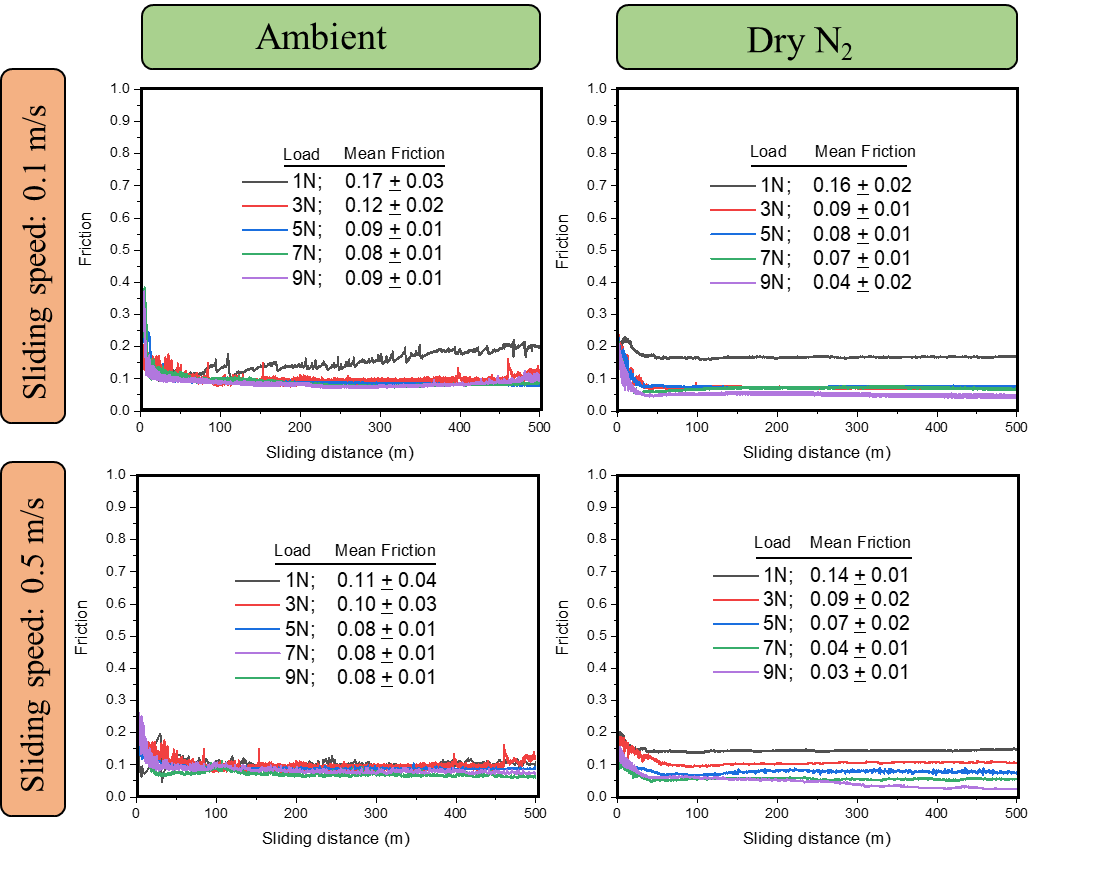


Supplemental Figure 2: Friction observed on the different tribopair systems in ambient air and in the dry nitrogen environments.

All the friction tests showed a short run-in period (also referred to as break-in period). This short duration was accompanied by the shearing of the 2D nanomaterials and forming a stable lubricious tribolayer that facilitated the reduction in friction. The initial phase was always characterized by a decreasing trend indicating the instantaneous on-set of shearing of MoS_2_ and GO. However, one isolated exception is in the case of 1 N 0.1 m/s sliding in dry nitrogen. There is indeed a very small rise in friction, after which it follows the classical run-in behavior. This behavior has its origin the stochastic contact conditions. In a condition where the ball makes a contact with a large MoS_2_-GO particle, the load sensor momentarily experiences resistance while compressing it. This is observed as increased friction during initial sliding. Upon the shear deformation of the local phase, run-in sets in, which causes a reduction resistance, and there on transition into steady state friction. This is in line with the state-of-the-art understanding of the run-in behavior (Blau, P. J. On the nature of running-in. *Tribol. Int.* **38**, 1007–1012 (2005)).


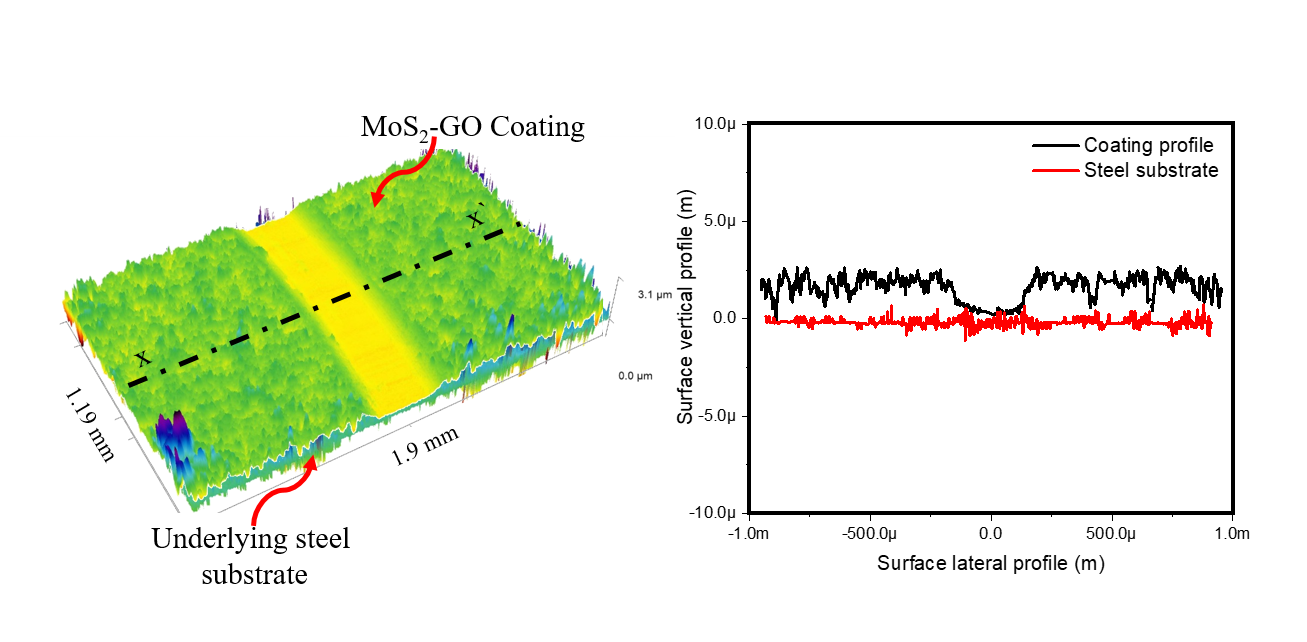


Supplemental Figure 3: The surface topography of the coated surface that of underlying steel and corresponding line profiles are shown. It is clear from the two images that entire “surface deformation” of the coating was well within the coating thickness itself. It corroborates well with the SEM image (Figure 6) where we see no deformation or wear scar on the underlying steel. Wear as we traditionally understand, is material removal due to abrasion, adhesion, three-body or other such materials’ interaction. In this case, it is not wear-volume-loss in that sense, as no material is “lost” or removed. The as-deposited powdery coating is compacted and densified to form a robust tribofilm that provides excellent protection against wear and frictional losses.


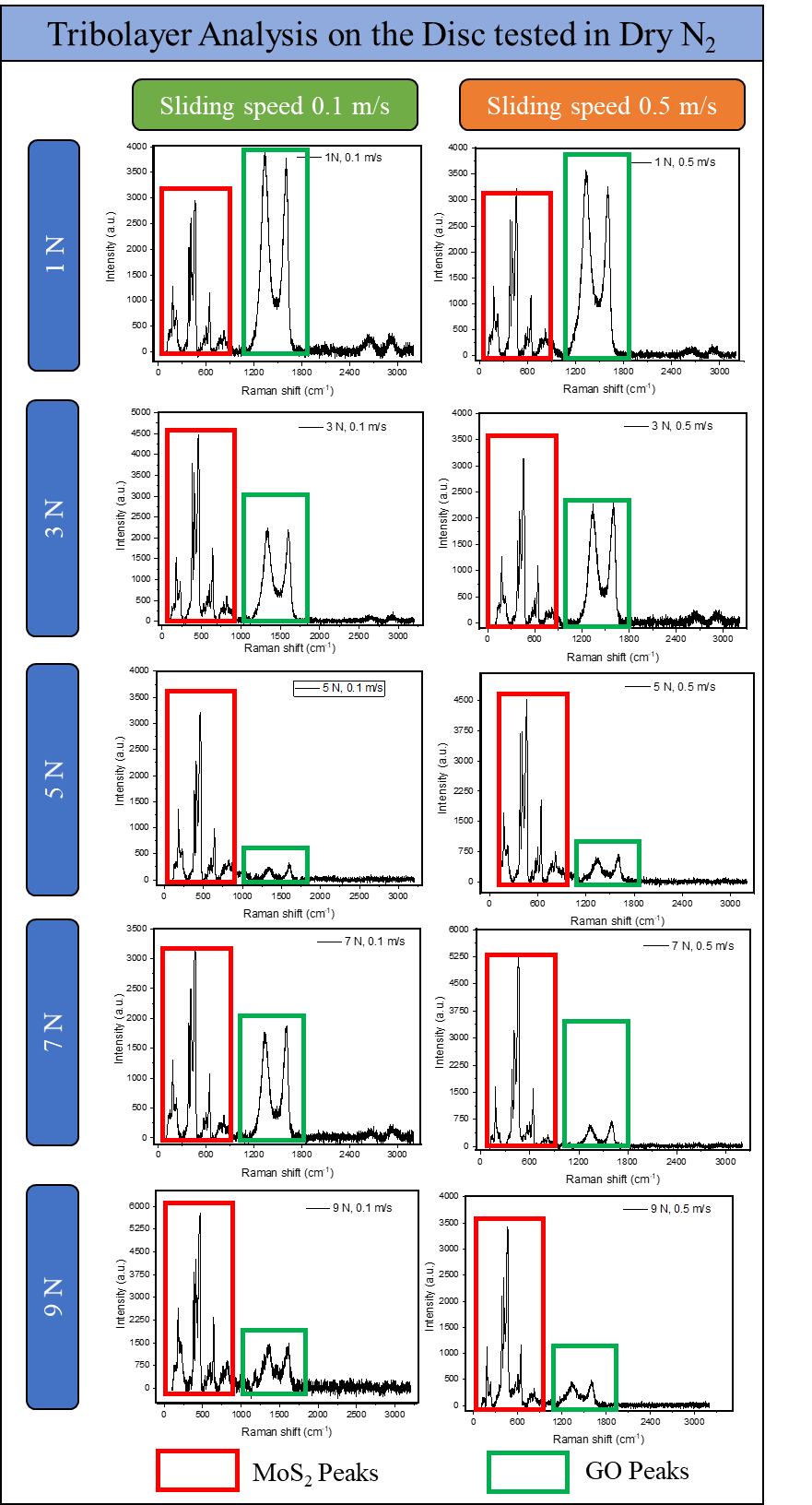


Supplemental Figure 4: Raman spectra of acquired on the discs tested in dry nitrogen


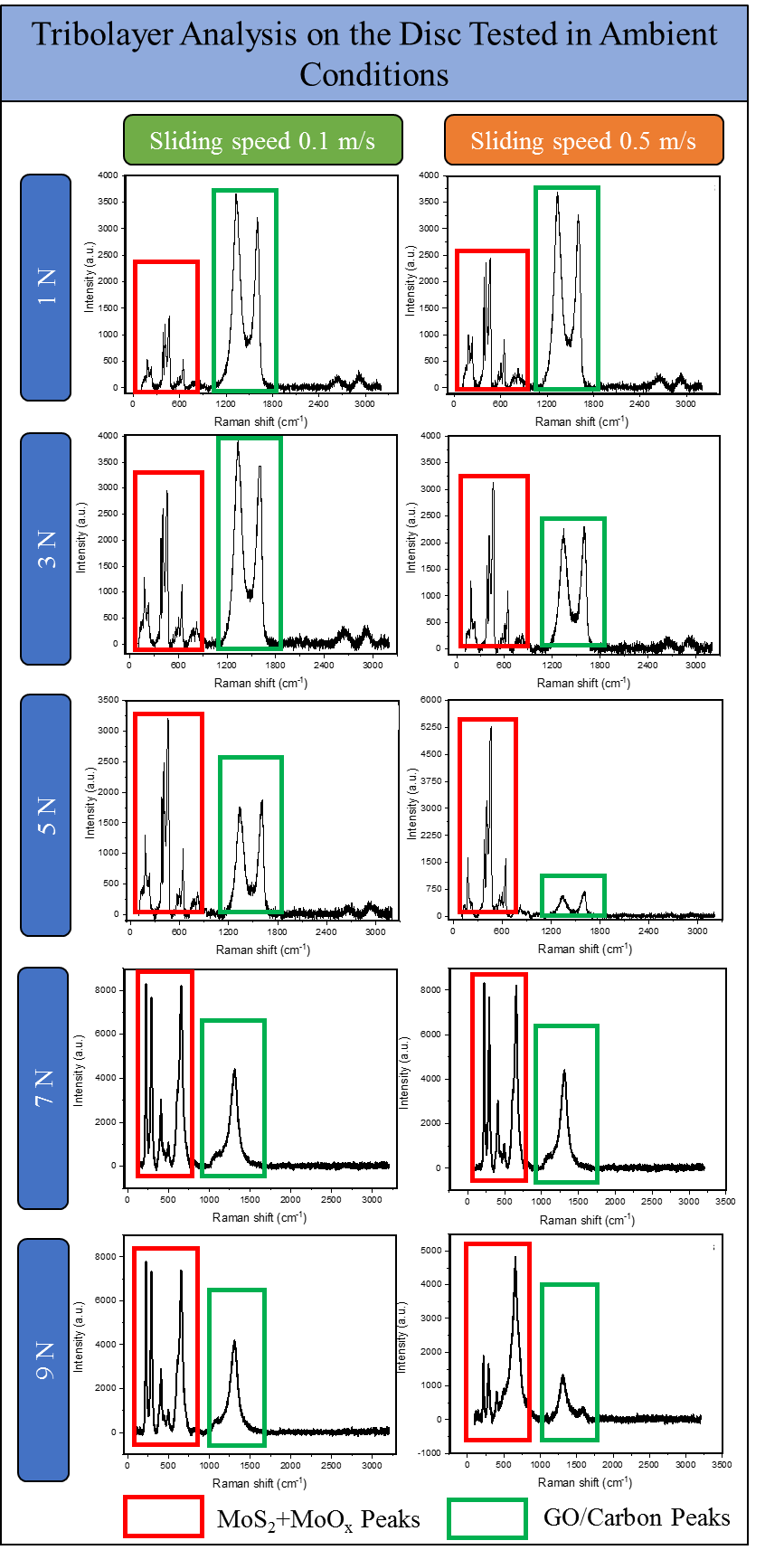


Supplemental Figure 5: Raman spectra of acquired on the discs tested in air
